# Virus-induced gene silencing in the perennial woody* Paeonia ostii*

**DOI:** 10.7717/peerj.7001

**Published:** 2019-05-29

**Authors:** Lihang Xie, Qingyu Zhang, Daoyang Sun, Weizong Yang, Jiayuan Hu, Lixin Niu, Yanlong Zhang

**Affiliations:** College of Landscape Architecture and Arts, Northwest A&F University, Yangling, Shaanxi, China

**Keywords:** *Paeonia ostii*, Virus-induced gene silencing, *Tobacco rattle virus*, Phytoene desaturase, Green fluorescent protein

## Abstract

Tree peony is a perennial deciduous shrub with great ornamental and medicinal value. A limitation of its current functional genomic research is the lack of effective molecular genetic tools. Here, the first application of a *Tobacco rattle virus* (TRV)-based virus-induced gene silencing (VIGS) in the tree peony species *Paeonia ostii* is presented. Two different approaches, leaf syringe-infiltration and seedling vacuum-infiltration, were utilized for *Agrobacterium*-mediated inoculation. The vacuum-infiltration was shown to result in a more complete *Agrobacterium* penetration than syringe-infiltration, and thereby determined as an appropriate inoculation method. The silencing of reporter gene *PoPDS* encoding phytoene desaturase was achieved in TRV-*PoPDS*-infected triennial tree peony plantlets, with a typical photobleaching phenotype shown in uppermost newly-sprouted leaves. The endogenous *PoPDS* transcripts were remarkably down-regulated in VIGS photobleached leaves. Moreover, the green fluorescent protein (GFP) fluorescence was detected in leaves and roots of plants inoculated with TRV-GFP, suggesting the capability of TRV to silence genes in various tissues. Taken together, the data demonstrated that the TRV-based VIGS technique could be adapted for high-throughput functional characterization of genes in tree peony.

## Introduction

Tree peony is a perennial woody plant belonging to sect. *Moutan* DC. of the genus *Paeonia* L. (Paeoniaceae) (Li, Hui & Wang, 2009). It is indigenous to China and the cultivation history can be traced back to 2000 years ago (Cheng, Li & Yu, 1998). As China’s unofficial national flower, tree peony has been introduced to Japan, America, Australia, and Europe, with a rise in worldwide popularity. It is commonly known as an ornamental and medicinal crop due to large showy flowers and abundant bioactive substances in roots. Recent reports suggest that the tree peony seed has high yield of oil which contains over 90% unsaturated fatty acids required by human, revealing a tremendous potential of tree peony in future edible oil production (Wu et al., 2014). The variety *Paeonia ostii* ‘Feng Dan’ (*P. ostii* ‘Feng Dan’) is a new oil crop widely planted in north China, with its total cultivated area exceeding 16,200 hectares.

For now, a quantity of studies have been carried out on the cloning and function analysis of genes, associated with flower development (Li et al., 2016), bud dormancy (Zhang et al., 2015b), anthocyanin accumulation (Zhang et al., 2015a), and fatty acid biosynthesis (Yin et al., 2018), in tree peony. However, the conclusive studies on the function of genes in tree peony are tough because the lack of efficient genetic transformation system. Besides, the transgenic technology is time-consuming and laborious for the generation of homozygous lines, especially for plants with long life cycle like tree peony.

Virus-induced gene silencing (VIGS) is an attractively quick strategy for reverse genetic manipulation of non-model plants bypassing the stable transformation process (Ruiz, Voinnet & Baulcombe, 1998; Burch-Smith et al., 2004). The VIGS experiment relies on the recombinant virus vector carrying an inserted partial sequence of a target plant gene to initiate RNA-mediated post-transcriptional gene silencing (PTGS), leading to transcript suppression of corresponding homologous gene (Baulcombe, 1999; Burch-Smith et al., 2004; Dinesh-Kumar et al., 2011). In this mechanism, double-stranded chimeric intermediates are first formed during viral replication in plant. Theses foreign intermediate are recognized and cleaved into 21–23 nucleotides of short interfering RNAs (siRNAs) by the enzyme DICER. Next, siRNAs are incorporated into the RNA-induced silencing complex (RISC) and target the complementary transcripts for cleavage, thus resulting in a specific degradation of host mRNA (Bartel, 2004; Senthilkumar & Mysore, 2011). In contrast to gene silencing methods with inverted repeat sequences, VIGS has several advantages as simple plasmid assembly, short implementation cycle, and available identification of embryo-lethal genes (Reid, Chen & Jiang, 2009).

Many viral vectors have been developed for VIGS assay, including *Apple latent spherical virus* (ALSV), *Barely stripe mosaic virus* (BSMV) (Holzberg et al., 2002), *Cucumber mosaic virus* (CMV) (Tasaki et al., 2016), *Potato virus X* (PVX) (Faivrerampant et al., 2004), *Tobacco mosaic virus* (TMV) (Kumagai et al., 1995), and *Tobacco rattle virus* (TRV) (Ratcliff, Martin-Hernandez & Baulcombe, 2001). Compared to other viruses, TRV is capable of reaching apical meristem, inducing mild symptoms, and infecting wide range of plant species. Consequently, TRV vector has been widely used for silencing genes in a number of eudicots and monocots (Purkayastha & Dasgupta, 2009), such as Arabidopsis (Burchsmith et al., 2006), tobacco (Liu, Schiff & Dineshkumar, 2002), tomato (Quadrana et al., 2011), petunia (Sun et al., 2017), strawberry (Jia et al., 2011), rose (Wu et al., 2016), gladiolus (Singh et al., 2013), wheat, and maize (Zhang et al., 2017). At present, the VIGS technique is mostly applied to small herbaceous plants, and only a minority of woody plants achieves the set-up of VIGS system, like physic nut (Ye et al., 2009), grape (Kurth et al., 2012), and apple (Yamagishi & Yoshikawa, 2013). The previous evidences indicate that tobacco rattle virus has been found in peony (*Paeonia lactiflora* ‘Sarah Bernhardt’) (Robertson et al., 2009). However, whether TRV-based VIGS can be applied to tree peony remains largely unknown.

Reporter gene is an essential component for indicating sites of silencing in VIGS system. PHYTOENE DESATURASE (PDS) is a key enzyme in the biosynthesis of protective carotene (Cunningham & Gantt, 1998). Silencing of *PDS* results in characteristic photobleaching symptoms in infected plants (Di Stilio et al., 2010), and therefore it usually serves as a clear reporter. A modified TRV-GFP vector, bearing the coding region of enhanced green fluorescence protein (EGFP), also provides a visual tool for monitoring virus spread and silencing efficiency. This vector has been successfully tested in several plants, including Arabidopsis, tobacco, rose, strawberry, and chrysanthemum (Tian et al., 2014). In this study, we established an effective VIGS system in *P. ostii* triennial seedlings by vacuum infiltration of TRV-*PoPDS* and TRV-GFP. The upper systemically-infected leaves with TRV-*PoPDS* displayed a prominent photobleaching phenotype and decreased *PoPDS* transcripts. GFP fluorescence was observed in TRV-GFP-infiltrated leaves and roots under UV light irradiation. The data we have obtained demonstrated the value of TRV-based VIGS for unraveling the functional significance of genes in tree peony.

## Materials and Methods

### Plant materials and growth conditions

**Figure 1:**
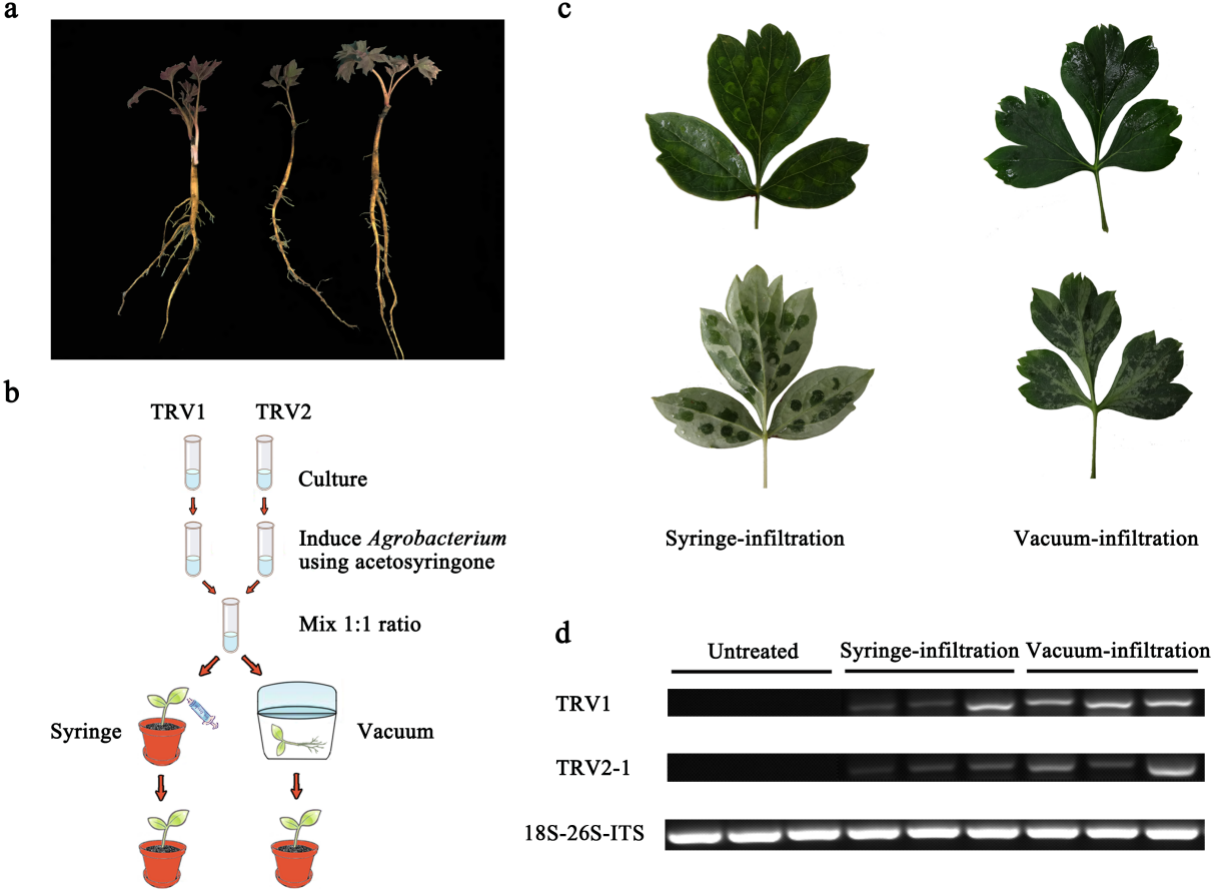
Comparison of syringe-infiltration and vacuum-infiltration methods with the TRV empty vector. (A) Three-year-old *P. ostii* plantlets at 4 weeks post germination used for agro-infiltration. (B) Schematic depiction of *Agrobacterium*-mediated TRV inoculation in *P. ostii* plants using syringe and vacuum methods. (C) The *P. ostii* leaves subjected to syringe- and vacuum-infiltration with TRV empty vector. (D) Semi-quantitative RT-PCR analysis of TRV1 and TRV2-1 accumulation levels in TRV empty vector-inoculated leaves by syringe and vacuum methods. 18S-26S internal transcribed spacer (18S-26S ITS) was used as internal standard.

Three-year-old seedlings of tree peony (*P. ostii* ‘Feng Dan’) at four weeks post germination were used for VIGS assay (Fig. 1A). The whole plant and leaves were agro-infiltrated with disposable syringe and vacuum pressure for infection of TRV constructs, respectively. After inoculation, the tree peony seedlings were rinsed with distilled water once and planted into plastic pots containing a mixture of peat moss and vermiculite in a 3:1 volume ratio. Those plants were first kept in the dark room at 15 °C for one week, and then transferred into a growth chamber with a 16 h light/ 8 h dark photoperiod, and a day/night temperature range of 20/18 °C. The inoculated and uppermost systemically-infected leaves were used for phenotype observation, expression profile analysis, and GFP fluorescence detection.

### Isolation and sequence analysis of *PoPDS*

Total RNA was extracted from the *P. ostii* ‘Feng Dan’ leaves with the TIANGEN RNA Prep Pure Plat kit according to the manufacturer’s recommendations (Tiangen, China). The first strand of cDNA was synthesized using PrimeScript® RT reagent Kit with gDNA Eraser (Takara, Shiga, Japan). Primers were designed to amplify the *PoPDS* coding sequence based on transcriptome data during leaf development of *P. suffruticosa* Andrews (Luo et al., 2017). PCR was conducted using Taq DNA polymerase (Invitrogen, USA). The PCR reaction procedure was as follows: a cycle of 94 °C for 5 min; 35 cycles of 94 °C, 30 s, 54 °C, 30 s, 72 °C, 30 s; a final cycle of 72 °C for 10 min. Next, the PCR products were cloned into the pUCm-T vector (TaKaRa, Shiga, Japan). Positive clones were confirmed by DNA sequencing. Corresponding amino acids were deduced through the ExPASy translate tool (http://web.expasy.org/translate/). Multiple sequence alignment of PoPDS with other similar proteins was performed by CLUSTALW (http://www.genome.jp/tools/clustalw/).

### Plasmid construction

The TRV1, TRV2, and TRV2-GFP plasmids were kindly provided by Dr. Yule Liu (Tsinghua University, China). To generate the TRV-*PoPDS* construct, a 195-bp *PoPDS* fragment was PCR-amplified using specific primers (Table 1), and cloned into the pUCm-T vector by T4 DNA ligase (Sangon, Jiangsu, China). This recombinant plasmid was digested with *Bam* HI and *Eco* RI restriction enzymes, and the fragment of *PoPDS* (GenBank accession number: mK733916) was ligated into corresponding sites of TRV2 vector (Fig. S2). The resulting construct was then transformed into *Eacherichia coli* strain DH5 *α* competent cells, which were selected on LB plates containing 50 mg l^−1^ of kanamycin. PCR was used to examine the presence of *PoPDS* insert in the generated construct.

**Table 1:** Primers used for RT-PCR amplification and construction of recombinant TRV2 plasmids.

Primer name	Nucleotide sequence (5′-3′)	Purpose	Size
PoPDS-F1	TCGGAGTTGGGTTCGCTGC	Cloning of *PoPDS* coding region	1,797 bp
PoPDS-R1	ATTCTGATGTGTTTTGTAGCC		
PoPDS-F2	CAGCCGATTTGATTTCCTTG	Cloning of inserted fragment for VIGS	195 bp
PoPDS-R2	CCTTGTTTTCTCATCCAGTC		
PoPDS-F3	AGTCATTGGGGGGTCAGGTCCG	RT-PCR	312 bp
PoPDS-R3	CAGCATACACACTCAGAAGGGG		
TRV1-F	CAGTCTATACACAGAAACAGA	TRV1-RNA detection	463 bp
TRV1-R	GACGTGTGTACTCAAGGGTT		
TRV2-1F	GGCTAACAGTGCTCTTGGTG	TRV2-RNA detection	359 bp
TRV2-1R	GTATCGGACCTCCACTCGC		
TRV2-2F	CGAGTGGAGGTCCGATACG	TRV2-RNA (containing inserted fragment) detection	Depending on insert
TRV2-2R	CGGTTCATGGATTCGGTTAG		
GFP-F	ATGGCCAACACTTGTCACTACTT	GFP-RNA detection	260 bp
GFP-R	ATTCCAATTTGTGTCCAAGAATG		
18S-26S-ITS-F	ACCGTTGATTCGCACAATTGGTCA	RT-PCR	150 bp
18S-26S-ITS-R	TACTGCGGGTCGGCAATCGGACG		

### Agro-inoculation of TRV vector

TRV1, TRV2, and its derivatives were introduced into *Agrobacterium tumefaciens* strain GV3101 via freeze-thaw method (Yan et al., 2012). The transformed bacteria bearing TRV constructs were cultured in LB medium supplemented with 40 mg l^−1^ kanamycin, 20 mg l^−1^ gentamicin, 10 mM MES, and 20 µM acetosyringone at 28 °C in a growth chamber for 48 h. *Agrobacterium* cultures were centrifuged at 4,000 *g* for 20 min, and resuspended in the infiltration buffer (10 mM MgCl_2_, 10 mM MES, and 200 µM acetosyringone) to a final OD_600_ of 1.0. The cultures containing TRV1 and TRV2 constructs was shaken gently for 4–6 h at room temperature and mixed together in a 1:1 ratio before inoculation. For syringe infiltration, the abaxial sides of two or three fully expanded leaves were injected using a 1-ml needleless syringe. For vacuum infiltration, the whole plants were submerged in the infiltration buffer and subjected to 0.1 MPa vacuum pressure for 20 min. Approximately 50 tree peony seedlings were inoculated by vacuum method for each assay.

### Semi-quantitative RT-PCR and quantitative real-time PCR

Total RNA was extracted from inoculated and systemically-infected leaves of tree peony seedlings, and purified with RNase-free DNase (Takara). First-strand cDNA as the template for PCR was synthesized from 2–5 µg of total RNA. Three primer pairs were designed to detect the presence of TRV (Table 1). Since the forward and reverse primers of TRV2-2 covered the multiple cloning sites (MCS), the size of resulting product varied depending on the inserts in the site, whereas the TRV1 and TRV2-1 primers led to the bands with the same sizes (Sun et al., 2016). The PCR products were analyzed through electrophoresis using a Molecular Imager Gel Doc XR+ System (Bio-Rad, Hercules, CA, USA). Quantitative real-time PCR (qRT-PCR) was carried out using SYBR Premix Ex Taq II (Takara) in a 20-µl PCR mixture and analyzed by a StepOnePlus Real-time PCR System (Applied Biosystems, Foster City, CA, USA). 18S-26S internal transcribed spacer was used as an internal control to normalize the expression data (Zhang et al., 2018). The PCR primers, used for the determination of transcript abundances of *PoPDS*, were designed outside the region of the inserted fragment to avoid amplification of the fragment included in TRV2 construct.

### GFP imaging

Transient assay of GFP in the inoculated leaf and root cells of *P. ostii* was conducted based on the agro-infiltration with TRV-GFP. GFP fluorescence was detected and photographed using a laser scanning confocal microscope (Leica TCS SP8).

### Western blot

A GFP-specific antibody (Abcam Inc., Cambridge, UK) was used to implement western blot analysis. Proteins were extracted from leaves and roots of *P. ostii* plants, with 300 µL extraction buffer (100 mM Tris pH = 6.8, 2.5% SDS, 100 mM dithiothreitol, 100 mM NaCl, and 10% glycerol). Bradford assay was used to determine protein quantities, and equal amounts of proteins for each sample were separated by 10% SDS-PAGE (Bradford, 1976). Next, proteins were transferred to a polyvinylidene difluoride membrane (GE healthcare). CP-GFP was detected after an overnight incubation at room temperature with a 1:10,000 dilution of the anti-GFP antibody conjugated to alkaline phosphatase (Tian et al., 2014). Alkaline phosphatase was detected using a chemiluminescent substrate (CSPD; Roche) and exposed to X-ray film (Kodak X-OMAT BT Film/XBT-1).

## Results

### Comparison of the agro-infiltration methods

In view of the woody characteristics of tree peony, choosing a plant with optimal age and size for VIGS assay is pre-requisite. Three-year-old young plantlets were therefore used because of their delicate underground roots, small plant type, and high occurrence of new leaves (Fig. 1A). To determine the most appropriate method of *Agrobacterium*-mediated TRV infection in tree peony, leaf syringe-infiltration and seedling vacuum-infiltration were selected for comparison (Fig. 1B). We found that the vacuum infiltration brought about a more sufficient permeation of bacterial cultures through the abaxial leaf surface than the syringe infiltration, which made the infiltration happen only at the inoculation sites. Furthermore, the syringe infiltration inevitably caused obvious mechanical damage to leaf tissues (Fig. 1C). Semi-quantitative RT-PCR analysis indicated that TRV1 and TRV2 transcripts were detected in all inoculated leaves by both infiltration methods, but not in untreated leaves (Fig. 1D). Also, the TRV transcripts accumulation levels in vacuum-infiltrated leaves were obviously higher than that in syringe-infiltrated leaves. According to the results, vacuum infiltration was used for subsequent gene-silencing experiments.

### Identification of *PoPDS*

*Phytoene desaturase* (*PDS*) is commonly used as a visible reporter for silencing. Based on the transcriptome data obtained from developing leaves of tree peony, we PCR-amplified the open reading frame (ORF) nucleotide sequence of *P. ostii PDS*, annotated as *PoPDS*. *PoPDS* was predicted to encode a protein of 575 amino acids, and conserved domain analysis revealed a putative dinucleotide binding domain in its deduced protein sequence. Multiple sequence alignments showed that amino acid sequence of *PoPDS* shared high similarity with the homologies from other plant species, such as *Vitis vinifera*, *Nicotiana tabacum*, *Arabidopsis thaliana*, and *Petunia hybrida* (Fig. S2). The full-length amino acid sequence of PoPDS had 83.3%, 82.09%, 79.96%, and 80.7% identities with those of four plant species, respectively.

### Silencing of *PoPDS* in *P. ostii* leaves

**Figure 2:**
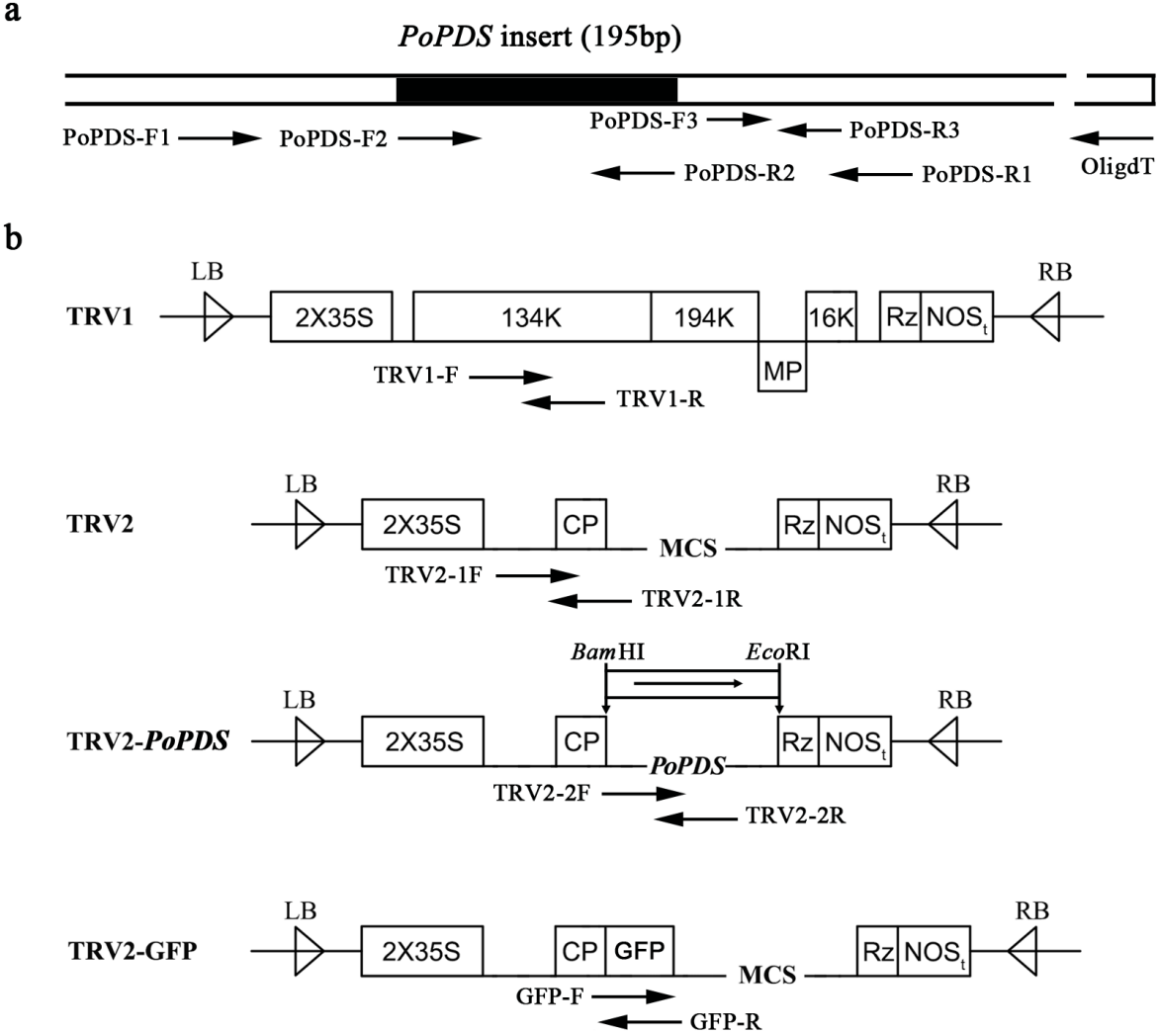
Schematic representation of TRV constructs used in this study. (A) The cDNA of *PoPDS* insert for its introduction into TRV vector. PoPDS-F1/PoPDS-R1 was used to amplify the open reading frame region of *PoPDS*, PoPDS-F2/PoPDS-R2 targeted the inserted fragment of*PoPDS* (the black box), and PoPDS-F3/PoPDS-R3 was designed for quantitative real-time PCR. (B) The structures of TRV1, TRV2, TRV2-*PoPDS*, and TRV2-GFP. The arrows indicate the different primer pairs for examining TRV1, TRV2-1, TRV2-2, and GFP transcript levels. LB left border, RB right border, MP movement protein, 16K 16 Kd protein, Rz self-cleaving ribozyme, NOSt NOS terminator, CP coat protein, MCS multiple cloning site.

**Figure 3:**
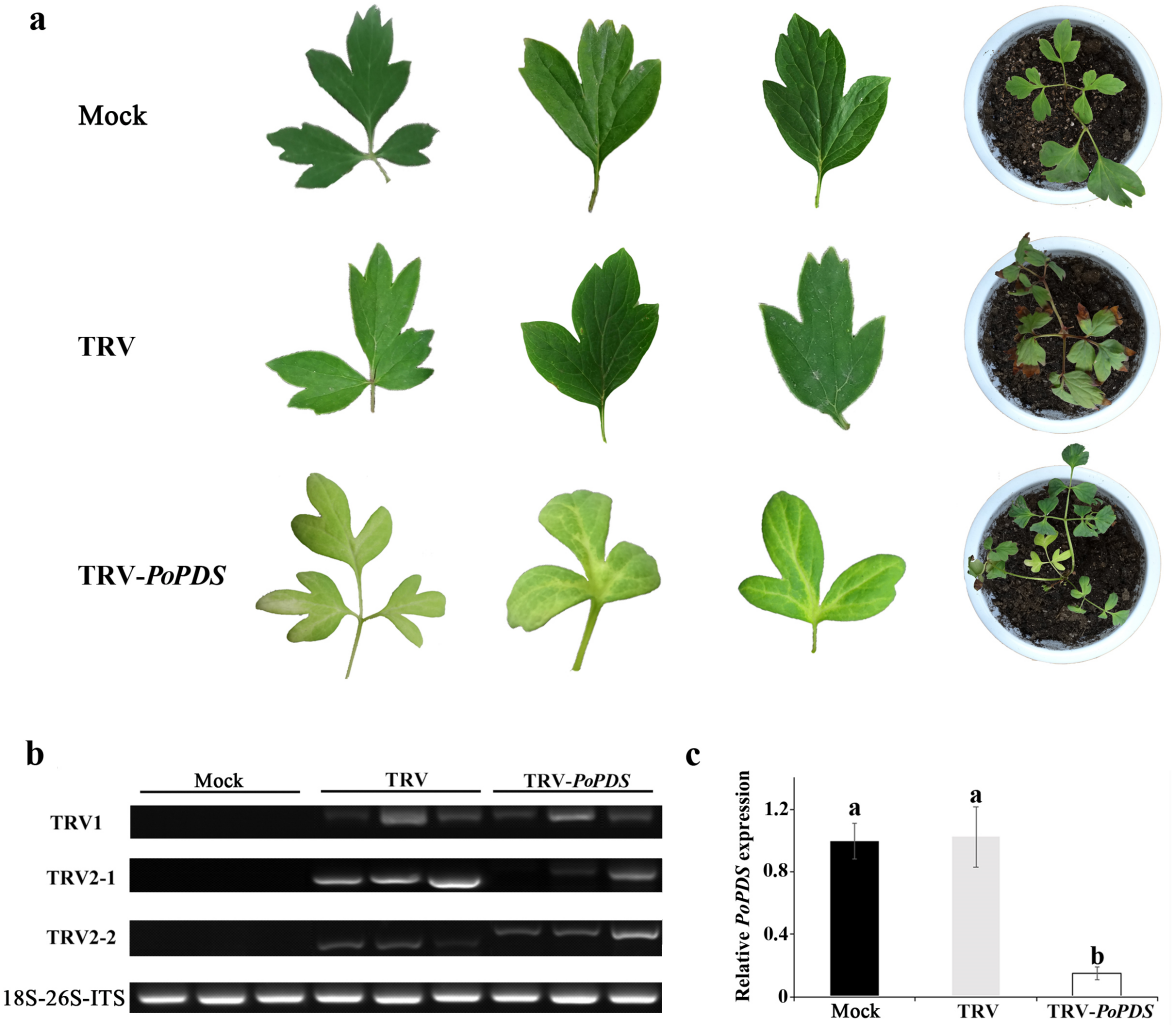
Silencing of *PoPDS* in *P. ostii* leaves infected with TRV-*PoPDS*. (A) Representative phenotypes of mock treated, TRV-(empty vector), and TRV-*PoPDS*-infected leaves in *P. ostii* seedlings. Photobleaching phenotypes were observed in the first newly-developed leaves of seedlings at 5 weeks post infiltration with TRV-*PoPDS*. (B) Semi-quantitative RT-PCR analysis of TRV1 and TRV2 accumulation levels in agro-infected *P. ostii* leaves. (C) Quantitative real-time PCR analysis of *PoPDS* in agro-infected *P. ostii* leaves. 18S-26S internal transcribed spacer (18S-26S ITS) was used to normalize the transcript levels, and relative expression values were calculated compared with the highest expression value taken as 1.0 (untreated). Error bars represent ± SE of data from three independent experiments. The different letters indicate significant differences using Duncan’s multiple range test at *p* < 0.05.

To assess the feasibility of TRV-based VIGS in tree peony, we introduced a 195-bp conserved fragment of *PoPDS* into TRV2 vector, and generated a TRV-*PoPDS* recombinant (Fig. 2). Upon *Agrobacterium*-mediated infection, similar necrotic symptoms occurred in the edge of leaves infiltrated with TRV empty vector and TRV-*PoPDS*, while the remaining area seems normal (Fig. 3A). Approximately 52% of seedlings exhibited a remarkable photobleaching phenotype in the first newly developed leaves at 4 weeks post inoculation. White spots or sectors were clearly observed throughout the upper leaves particularly around leaf main veins. This phenotype remained stable and persisted for about 2 months under growth chamber conditions (Fig. S3).

To confirm the correlation of leaf photobleaching with the presence of the viral vectors, TRV accumulation was examined using semi-quantitative RT-PCR. TRV1 and TRV2 were detected in TRV empty vector- and TRV-*PoPDS*-infected leaves, but not in the mock control plants (Fig. 3B). When using primers covering MCS of TRV2 vector, a fragment carrying *PoPDS* insert was detected in the leaves agro-infiltrated with TRV-*PoPDS*. 18S-26S internal transcribed spacer was referred as an internal control for normalization of gene expression. QRT-PCR analysis demonstrated that transcript abundances of *PoPDS* were significantly reduced in photobleached leaves of plants infiltrated with TRV-*PoPDS*, compared with that in mock- and TRV empty vector-inoculated seedlings (Fig. 3C). These results suggested that the leaf photobleaching phenotype was initiated by *PoPDS* silencing. It indicated that the *PoPDS* of tree peony could be silenced by VIGS and TRV infection was systemically established.

### Validation of TRV-GFP in *P. ostii* leaves and roots

**Figure 4:**
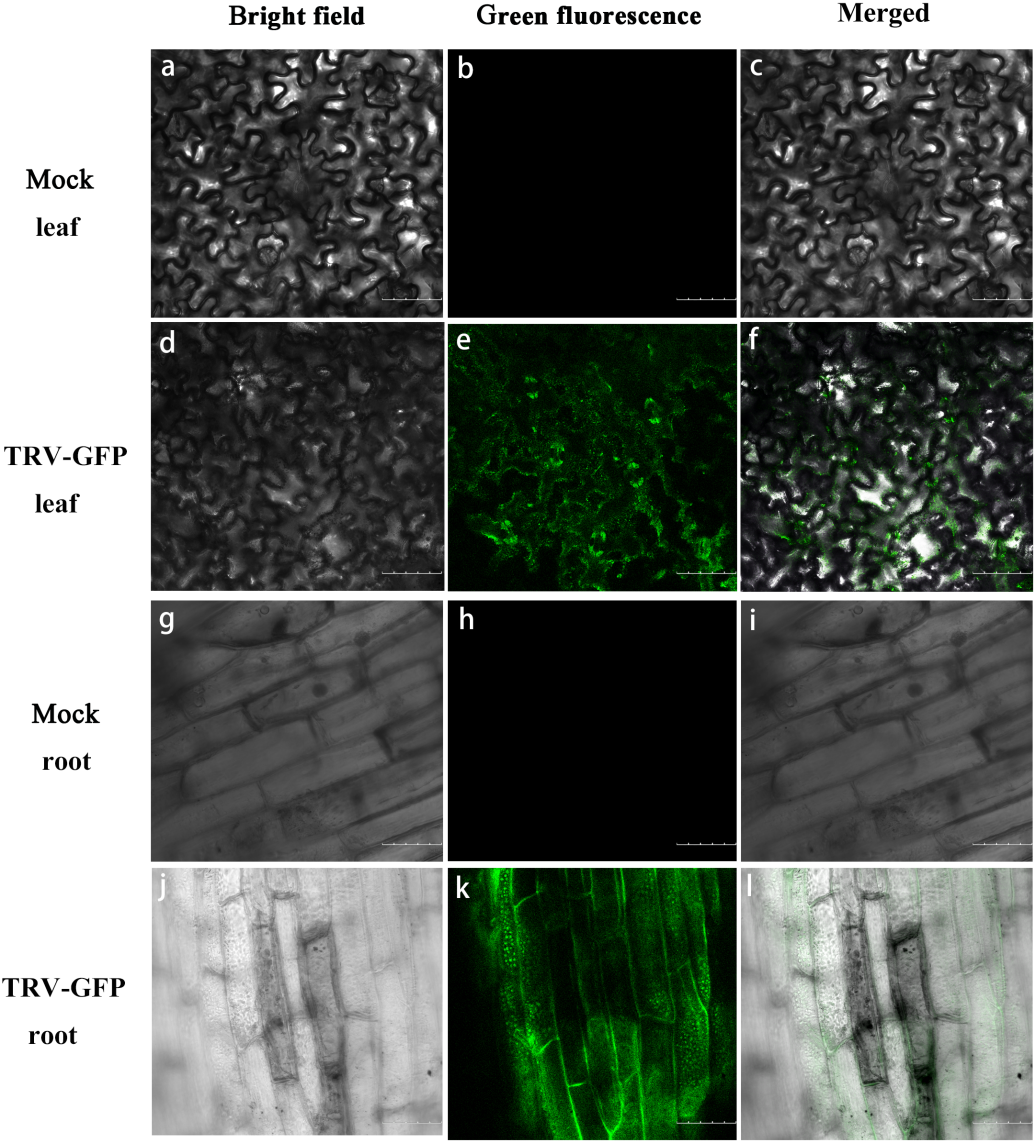
GFP expression in *P. ostii.* leaves and roots inoculated with TRV-GFP. Confocal microscopy image of *P. ostii.* leaves and roots infected with TRV-GFP at 5 days post-infiltration (dpi). Fluorescence was not observed in leaves (A–C) and roots (G–I) of mock-treated plants. The bright field (A, D, G, J), the GFP channel (B, E, H, K), and the merged images (C, F, I, L) of the bright field and the GFP channel are shown. Scale bars equal to 100 µm (A–F) or 75 µm (G–L).

Apart from TRV-*PoPDS*, another visualizable vector TRV-GFP, in which the EGFP coding sequence was fused to coat protein ORF of TRV2, was used for infiltration to monitor virus spread in *P. ostii*. Under a confocal microscope, GFP fluorescence was observed in the newly emerging leaves and roots of plants at 5 days post inoculation (dpi) with TRV-GFP, indicating the capability of TRV vector to express foreign genes in different tree peony tissues. No fluorescence signals were detected in mock control leaves and roots (Fig. 4).

**Figure 5:**
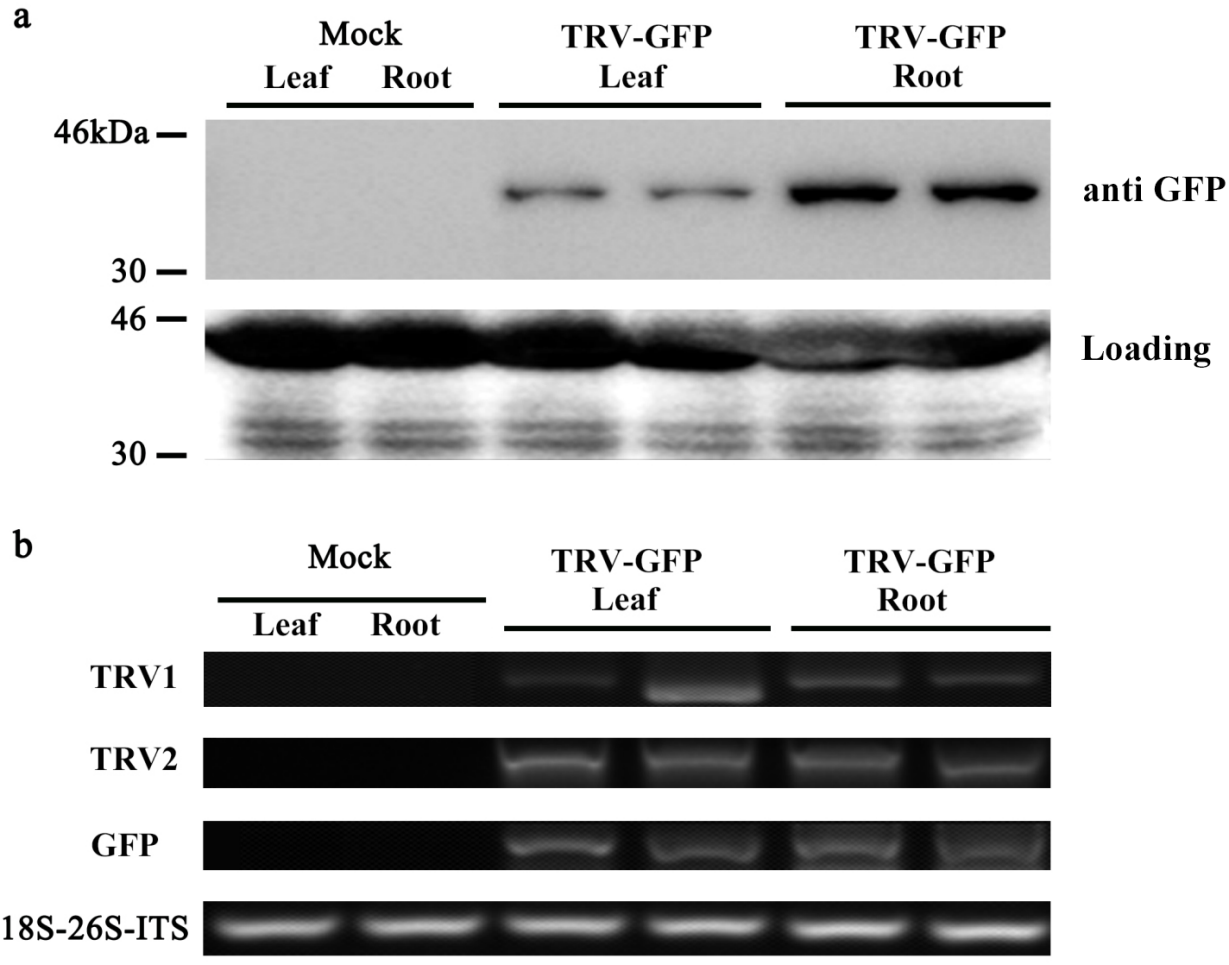
Detection of GFP protein accumulation in TRV-GFP-inoculated *P. ostii* leaves and roots. (A) Western blot analysis of CP-GFP protein levels in mock treated, TRV-GFP-infected *P. ostii* leaves and roots at 5 days post inoculation. Ten micrograms of protein were loaded into each lane and an anti-GFP antibody was used to detect the CP-GFP fusion protein. Coomassie blue staining was used to confirm equal loading in each lane. (B) Semi-quantitative RT-PCR analysis of TRV1, TRV2, and GFP accumulation levels in mock treated, TRV-GFP-infected *P. ostii* leaves and roots. 18S-26S internal transcribed spacer (18S-26S-ITS) was used as internal control.

Moreover, we performed western blot analysis to check the expression of GFP protein in infected leaves and roots. As illustrated in Fig. 5A, GFP proteins were accumulated in the leaves and roots of plants inoculated with TRV-GFP, whereas no GFP bands were found in control plants. By contrast, the GFP abundances in infected roots appeared to be much higher than that in infected leaves (Fig. 5A). Semi-quantitative RT-PCR analysis revealed a consistent variance that the roots exhibited more transcripts of TRV1, TRV2, and GFP than the leaves (Fig. 5B). The data suggested that the systemic movement of TRV vector in tree peony plants could be effectively supervised via the GFP-tagged expression.

## Discussion

In addition to the significant floral characteristics of tree peony, its roots contains some special secondary metabolites, which are generally used as traditional Chinese medical materials, and its leaves also have excellent ornamental value owing to their changeable color during the early growth period (Luo et al., 2017; Li et al., 2018). Therefore, there are considerable interests in evaluating the gene function in both roots and leaves of tree peony. However, an effective genetic transformation system is still unavailable in tree peony because of severe callus browning and tough plant regeneration (Liu & Jia, 2010). Few studies on molecular functional identification have been performed in tree peony due to this limitation. It seems likely that a transient expression system for up- or down-regulation of genes in tree peony would be greatly needed.

The VIGS technique has been widely used in various plant species as a rapid, convenient, and efficient tool for functional assessment of genes (Wege et al., 2007; Velásquez, Chakravarthy & Martin, 2009). In the present study, whether TRV-based vector could be used for silencing endogenous genes in tree peony was investigated. Our results demonstrated that the conventional leaf syringe-infiltration method is laborious and it resulted in an inadequate infiltration to *P. ostii* leaves, when compared with seedling vacuum-infiltration. It is quite likely that the physiological structure of tree peony leaf affected the entering of agrobacterial mixture. Not many stomatal apparatus existed in the lower epidermis of tree peony young leaf, and its leaf mesophyll cells were divided into a large number of vein islets by reticulate vein networks. Only a limited area of leaf could be effectively infiltrated with TRV constructs via syringe injection. Additionally, the thin tree peony leaves were prone to suffer mechanical damage from syringe-infiltration method. Previous studies also showed that the vacuum approach was more effective than other infiltration methods in woody plants (Ye et al., 2009; Liu et al., 2014). Thus, a vacuum-infiltration into the whole plant is probably considered as a good choice, when it comes to species that are difficult to infect.

Concerning the experimental materials for inoculation, it is well known that tree peony has a long juvenile stage that commonly lasts for about 3 years, during which the root is the main growing part (Wang et al., 2015). This development feature confined the application of VIGS on tree peony plants. Three-year-old seedlings of tree peony were consequently selected as agro-inoculated objects in our work. Since the plants at this stage were favorable to vacuum infiltration in size, and on the other hand to sprouting of upper new leaves. A visual silencing phenotype of marker gene, such as *PDS*-silenced leaf photobleaching or *chalcone synthase* (*CHS*)-silenced white-corollas phenotypes, requires upspring of systemically-infected tissues. Our results proved that a significant gene silencing took place in newly-developed leaves of triennial *P. ostii* plants. Because no reproductive buds were formed at this stage, a trial of gene silencing in tree peony floral organs via VIGS will be made in future work.

*PDS* has been frequently used as an indicator gene in VIGS systems because the silencing of *PDS* reduces photoprotective carotenoid levels in green tissues and thereby leads to chlorophyll photooxidation and tissue bleaching (Kumagai et al., 1995). In this study, we cloned the *PDS* gene from *P. ostii* leaves and constructed the TRV-*PoPDS* vector to unravel the function of *PoPDS* and verify the possibility of applying VIGS in tree peony. After infiltration with TRV-*PoPDS*, an expected silencing phynotype (photobleaching) was observed in systemically-infected leaves, while the directly inoculated leaves showed lesions resembling those of TRV empty vector. The results mentioned above indicated that a systemic TRV viral infection was established, and it was essential for the VIGS application. The silencing of *PoPDS* also demonstrated that TRV-based VIGS could be used as an effective method towards functional characterization of genes in tree peony plants.

It is noteworthy that almost all photobleached leaves resulting from TRV-*PoPDS* infection exhibited variegated phenotypes as white spots or sectors not completely white (Fig. 3A), and we hypothesized that multiple factors may contribute to it. The post-inoculation growth temperature largely influences the efficiency of VIGS-based gene silencing. It has been reported that low temperature enhances gene silencing efficiency when TRV-mediated VIGS is employed in tomato (Fu et al., 2006). But a conflicting finding is that low temperature suppresses gene silencing through the prevention of siRNA formation in *N. benthamiana* (Szittya et al., 2003). The length of inserted fragment in viral vector is also closely associated with gene silencing efficiency. As reported previously, different lengths of *PDS* inserts result in varied photobleaching patterns and ranges in TRV-infected tobacco (Liu & Page, 2008; Ye et al., 2009). Our VIGS procedure hence requires further optimization in temperature and inserted fragment size in future work. Furthermore, the *PoPDS*-silenced phenotypes were particularly significant along the leaf vein (Fig. 3A). It is in agreement with the results that viral propagation and/or systemic silencing response occur mainly along the vascular bundle system (Wege et al., 2007).

In order to visualize viral accumulation in infiltrated tree peony plants, the TRV-GFP vector was used. The GFP, a fluorescent protein from jellyfish (*Aequorea victoria*), does not participate in biological processes of plants. The gene was overexpressed driven by the 35S promoter and used as a marker to trace the presence of virus (Tian et al., 2014). In the present work, green fluorescence was observed in the roots and leaves of infected tree peony seedlings at 5 dpi, and the accumulation levels of TRV1, TRV2, and GFP were also detected (Fig. 4). The concentration of GFP protein was able to reflect the viral load and degree of silencing (Tian et al., 2014). Previous findings proved that TRV virus possesses the ability to move efficiently within the roots of infected plants (Macfarlane & Popovich, 2000). We found a higher transcript and protein levels of GFP in roots than that in leaves. It is concluded that virus infection may happen mainly in roots at first and then spread into newly-developed leaves after vacuum infiltration. Future work will examine the underlying mechanism for the discrepancy of TRV replication and movement in roots and leaves of tree peony. Altogether, it suggested that the TRV-GFP vector was available to tree peony plants and suitable for monitoring the systemic spread of TRV carrying target gene fragments. An advantage is that the employment of TRV-GFP construct could avoid the destruction of the photosynthetic apparatus caused by *PDS*-silenced leaf photobleaching.

## Conclusion

In conclusion, our results indicated that an effective TRV-based VIGS system was established in *P. ostii* based on TRV-*PoPDS* and TRV-GFP constructs. Seedling vacuum-infiltration was determined as an appropriate method for *Agrobacterium*-mediated infection of TRV, compared with leaf syringe-infiltration. A remarkable photobleaching phenotype was observed in TRV-*PoPDS*-infected upper new leaves, which was concomitant with substantial reduction in *PoPDS* transcripts. The detection of GFP fluorescence and accumulation levels in leaves and roots infected with TRV-GFP revealed TRV is a versatile tool to analyze gene function in different tissues of tree peony. Thus, this system we developed will be greatly helpful to characterize the function of genes associated with various molecular and physiological processes in tree peony.

##  Supplemental Information

10.7717/peerj.7001/supp-1 Supplemental Information 1 The open reading frame (ORF) nucleotide sequence of phytoene desaturase (PDS) of P. ostii * GenBank accession number of* PoPDS*: MK733916.*

10.7717/peerj.7001/supp-2 Figure S1 Uncropped blots used in this manuscript Full-length uncropped blots for Semi-quantitative RT-PCR analysis of TRV1 and TRV2-1 accumulation levels in TRV empty vector-inoculated leaves by syringe and vacuum methods (a-c); in systemically-infected *P.ostii* leaves (d-h), and TRV1, TRV2, and GFP accumulation levels in mock treated, TRV-GFP-infected *P. ostii* leaves and roots (i-k).

10.7717/peerj.7001/supp-3 Figure S2 Multiple sequence alignment of deduced amino acids of *PoPDS* with other homologies, including Vitis vinifera *VvPDS*, Nicotiana tabacum *NtPDS*, Arabidopsis thaliana *AtPDS*, and Petunia hybrida *PhPDS* Sequences were aligned using ClustalW program. The N-terminus and transient sequence and putative dinucleotide-binding domain are underlined. Red box denotes a conserved region in PDS protein sequences for VIGS. Black background represents identical amino acid residues. ** The GenBank accession number of *PoPDS* is MK733916.

10.7717/peerj.7001/supp-4 Figure S3 Phenotypes of TRV-*PoPDS*-infected leaves in *P. ostii* seedlings at 2 months after infiltration

## Additional Information and Declarations

### Data Availability

The following information was supplied regarding the deposition of DNA sequences: Data is available at GenBank (accession number: MK733916) and as a Supplemental File.
