# Supplementary material for: Virus-induced gene silencing in the perennial woody Paeonia ostii
Source: PeerJ. 2019 May 29;7:e7001. doi: 10.7717/peerj.7001 (PMC6545099; doi:10.7717/peerj.7001)
Supplement: Supplemental Information 1 — GenBank accession number of PoPDS: MK733916. [file peerj-07-7001-s001.docx]

Seq S1. The open reading frame (ORF) nucleotide sequence of *phytoene desaturase* (PDS) of *P. ostii.* GenBank accession number of *PoPDS*: MK733916.

ATGGCCCTTTATGGTTGTGTTTCCGCGGTGACGCCAACGCCAAGTAATAAAATCTCGCAATCCACCTTGACTCGTGGTTTTCGCATGAAAATCAATCCCGCGATGGCATTTGGAGATAGTGCTGCTATGGGTCTCAGCTTGAGAATTCCAAATACACACGCCATAACTACGAGGCCTAGAAAAGATGTCTTCCCTTTGCAGGTTGTTTGCGTGGACTATCCAAGACCAGAGCTTGACAATACTGTTAATTTCTTAGAAGCCGCTTACTTATCATCATTCTTCCGCTCTTCTTCCCGTCCAAATAAACCGTTGGATGTTGTGATTGCCGGTGCAGGTTTGGCTGGTTTATCAACTGCAAAATATTTAGCAGATGCAGGTCACAGACCTTTATTGTTGGAAGCAAGAGATGTTCTAGGTGGAAAGGTGGCTGCATGGAAAGATGACGATGGAGACTGGTATGAGACAGGGCTACATATATTCTTTGGGGCTTACCCAAATGTGCAGAACCTGTTTGGAGAACTTGGTATTAATGATCGGTTGCAGTGGAAGGAGCATTCTATGATATTTGCCATGCCTAACAAGCCAGGAGAATTCAGCCGATTTGATTTCCTTGAAGTACTGCCTGCACCTTTAAATGGCCTTTGGGCGATCCTGAAGAACAATGAAATGTTGACTTGGCCAGAAAAAGTGAAATTTGCGATTGGACTCTTGCCAGCAATTGTTGGCGGTCAGGCTTATGTTGAGGCTCAAGATGGTTTTACTGTTAAAGACTGGATGAGAAAACAAGGGATACCTGATCGAGTAACTAATGAGGTGTTTATTGCCATGTCAAAGGCACTAAACTTCATAAACCCAGATGAACTTTCAATGCAATGTATTTTGATTGCTTTGAACAGATTTCTTCAGGAGAAGCATGGTTCCAAGATGGCTTTCTTAGATGGCAATCCTCCAGAGAGACTCTGCATGCCAATTGTTGATCATATTGAGTCATTGGGGGGTCAGGTCCGTCTTAATTCAAGAATACAAAAGATTGAGTTGAATAAAGATGGAACCGTGAAGGGCTTTTTGCTTAATGATGGGAATTTAATTAAAGGAGATGCTTATGTATTCGCCACTCCAGTTGACATTCTGAAGCTTCTTCTGCCGAAAGAGTGGAAAGAGATTCCAGACTTTAAAAGACTGGAGAAGTTAGTTGGAGTTCCAGTTATAAATGTTCACATATGGTTTGACAGGAAGTTGAAGAACACATATGACCATTTACTTTTCAGCAGAAGTCCCCTTCTGAGTGTGTATGCTGACATGTCTGTAACTTGTAAGGAATATTACAACCCAAATGAATCTATGCTGGAGTTGGTTTTTGCTCCTGCAGAAGAATGGATCTCACGTAGTGACTCAGAAATTATTGATGCTACGATGAAAGAACTTGCAAAACTGTTTCCTGATGAAATTTCTGCGGATCAGAGCAAGGCTAAGATTTTGAAGTATCATGTTGTTAAAACACCGAGGTCCGTATATAAGACTGTCCCAGATTGTGAACCATGTCGTCCCTTACAAAGATCTCCAATAGAAGGATTCTATTTAGCAGGTGACTATACAAAACAGAAGTATTTGGCTTCTATGGAAGGTGCTGTTCTATCGGGAAAGTTTTGTGCACAGGCTATTGTGCAGGATTATGAATTGCTTGTTGCTCGGGAGCCGAAAAAATTGGCTGAGGTTCGCACCCTCTAA
