# Supplementary material for: Virus-induced gene silencing in the perennial woody Paeonia ostii
Source: PeerJ. 2019 May 29;7:e7001. doi: 10.7717/peerj.7001 (PMC6545099; doi:10.7717/peerj.7001)
Supplement: Figure S1 — Full-length uncropped blots for Semi-quantitative RT-PCR analysis of TRV1 and TRV2-1 accumulation levels in TRV empty vector-inoculated leaves by syringe and vacuum methods (a-c); in systemically-infected P.ostii leaves (d-h), and TRV1, TRV2, and GFP accumulation levels in mock treated, TRV-GFP-infected P. ostii leaves and roots (i-k). [file peerj-07-7001-s002.doc]

#
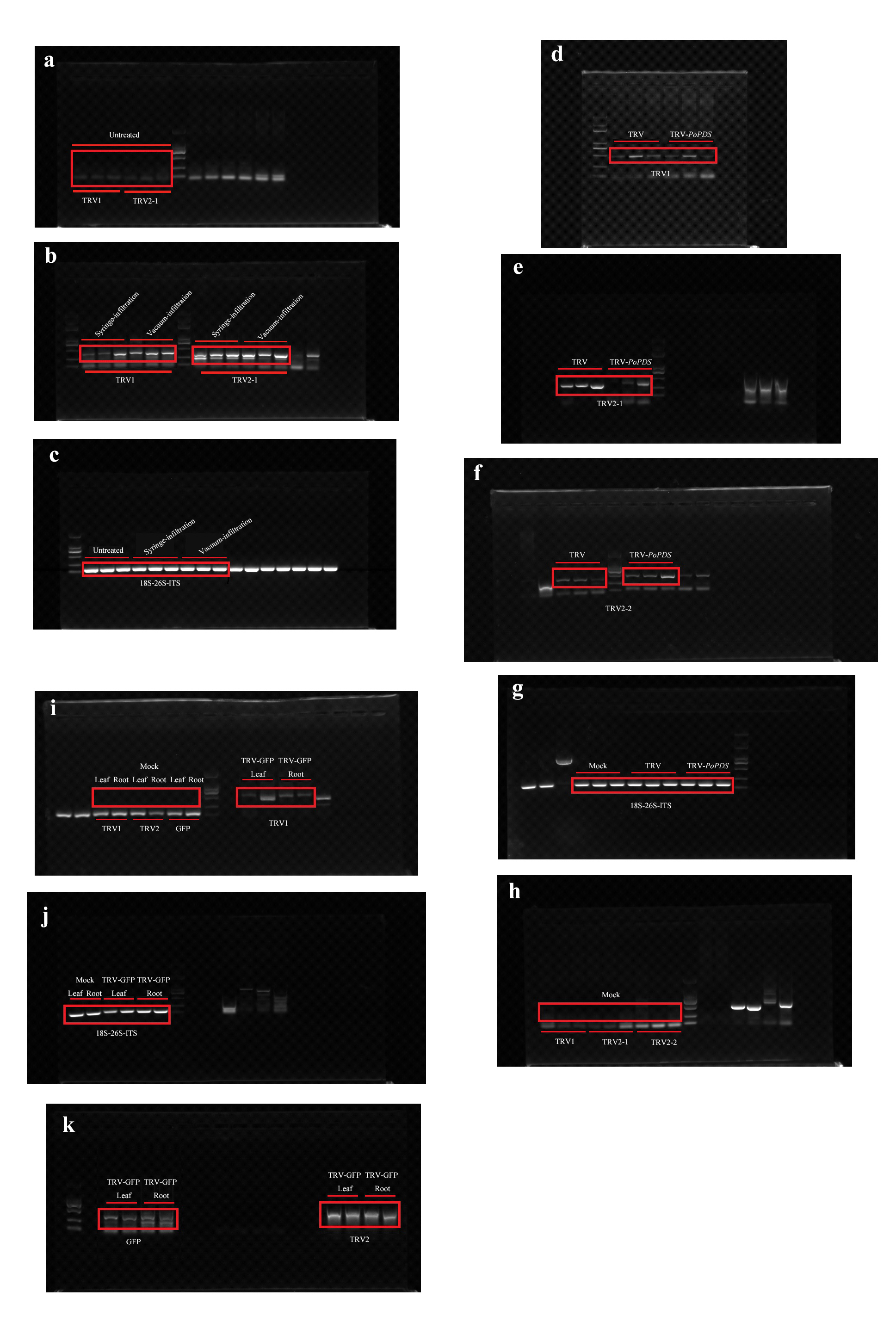


**Fig. S1. Uncropped blots used in this manuscript**

Full-length uncropped blots for Semi-quantitative RT-PCR analysis of TRV1 and TRV2-1 accumulation levels in TRV empty vector-inoculated leaves by syringe and vacuum methods (a-c); in systemically-infected P.ostii leaves (d-h), and TRV1, TRV2, and GFP accumulation levels in mock treated, TRV-GFP-infected P. ostii leaves and roots. (i-k)
