# Supplementary material for: Virus-induced gene silencing in the perennial woody Paeonia ostii
Source: PeerJ. 2019 May 29;7:e7001. doi: 10.7717/peerj.7001 (PMC6545099; doi:10.7717/peerj.7001)
Supplement: Figure S2 — Sequences were aligned using ClustalW program. The N-terminus and transient sequence and putative dinucleotide-binding domain are underlined. Red box denotes a conserved region in PDS protein sequences for VIGS. Black background represents identical amino acid residues. The GenBank accession number of PoPDS is MK733916. [file peerj-07-7001-s003.docx]

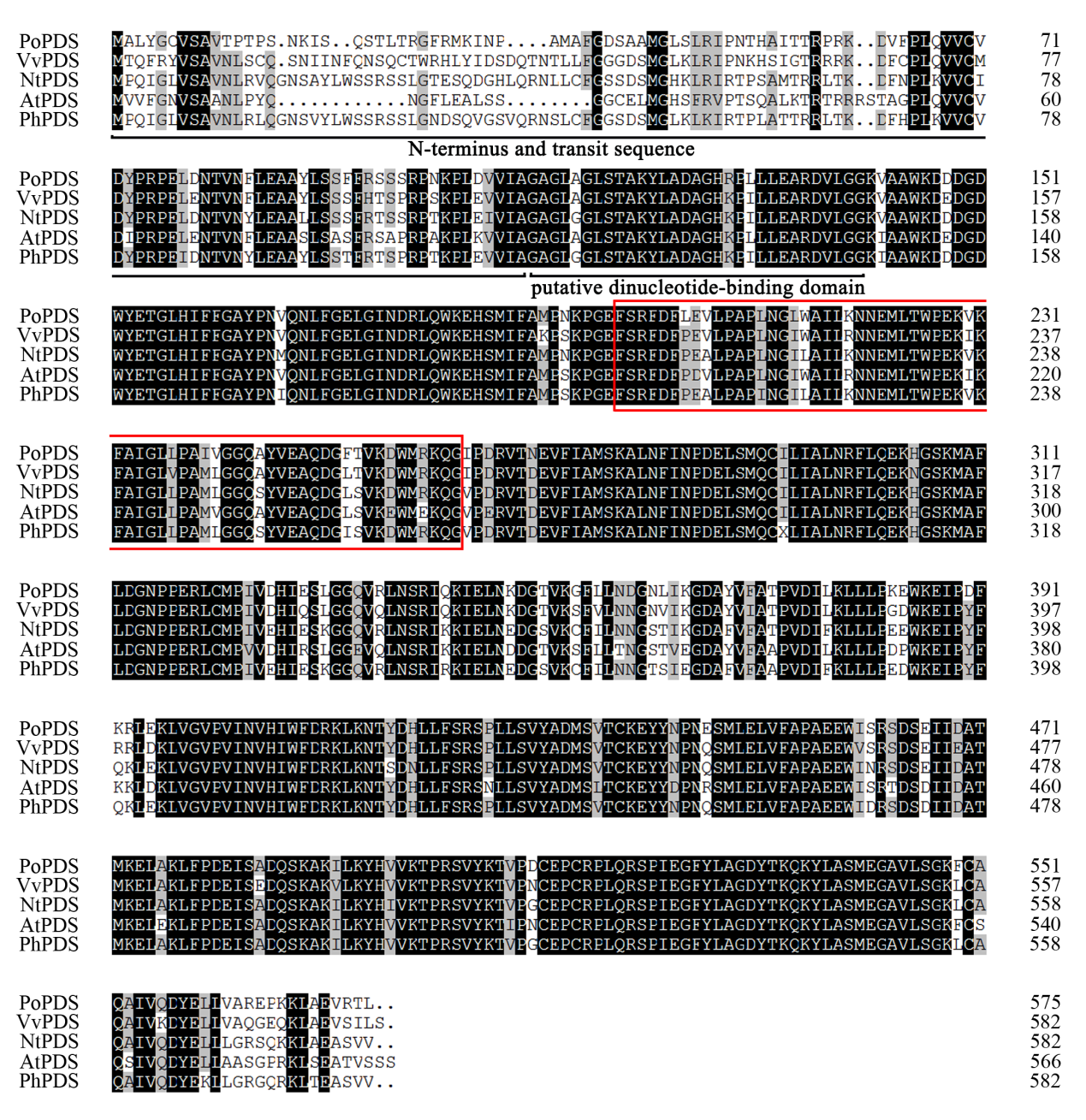


**Fig. S2 Multiple sequence alignment of deduced amino acids of *PoPDS* with other homologies, including Vitis vinifera *VvPDS*, Nicotiana tabacum *NtPDS*, Arabidopsis thaliana *AtPDS*, and Petunia hybrida *PhPDS***

Sequences were aligned using ClustalW program. The N-terminus and transient sequence and putative dinucleotide-binding domain are underlined. Red box denotes a conserved region in PDS protein sequences for VIGS. Black background represents identical amino acid residues. The GenBank accession number of *PoPDS* is MK733916.
