# Supplementary material for: Virus-induced gene silencing in the perennial woody Paeonia ostii
Source: PeerJ. 2019 May 29;7:e7001. doi: 10.7717/peerj.7001 (PMC6545099; doi:10.7717/peerj.7001)
Supplement: Figure S3 [file peerj-07-7001-s004.doc]

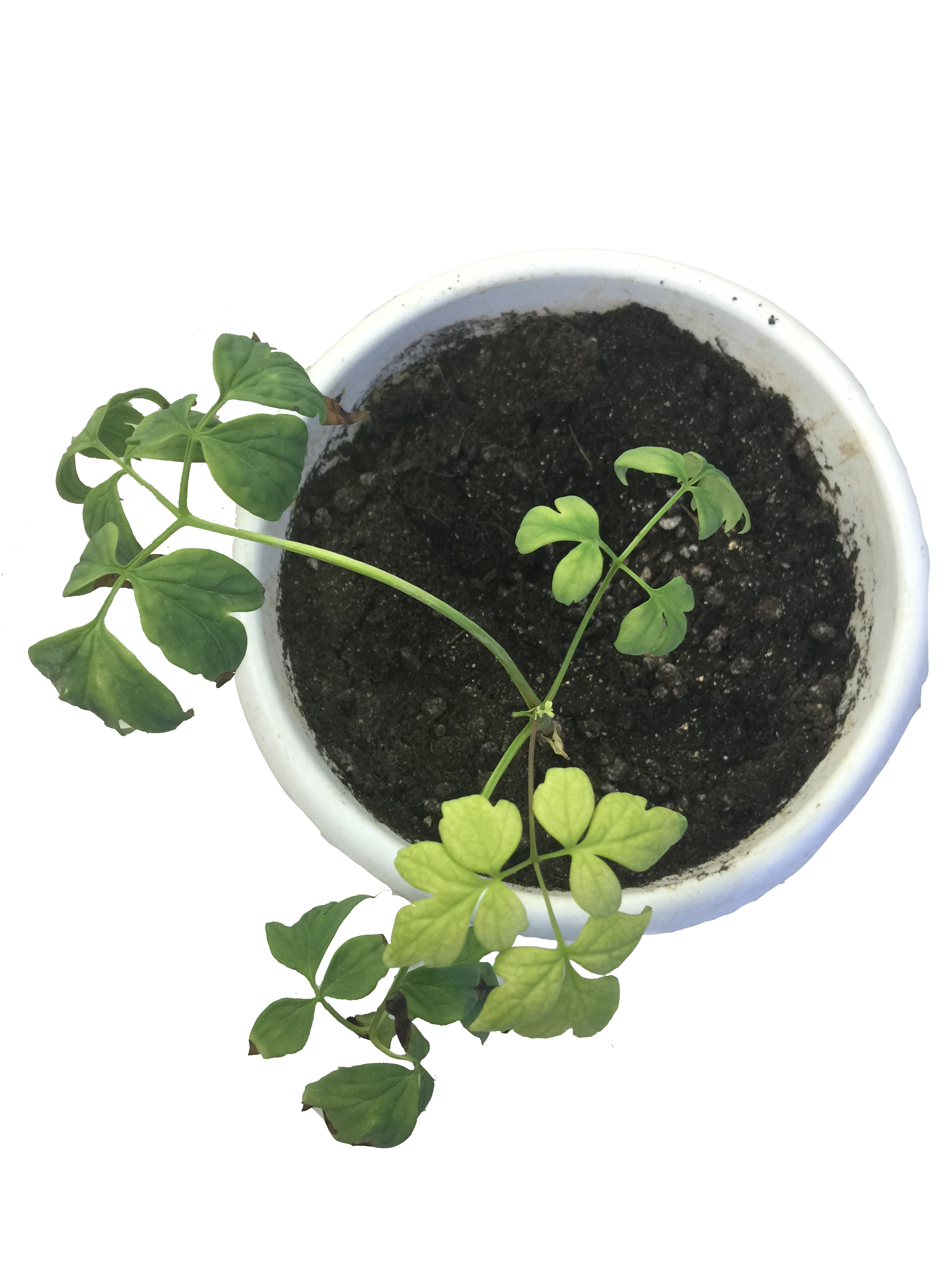


**Fig. S3 Phenotypes of TRV-*PoPDS*-infected leaves in *P. ostii* seedlings at 2 months after infiltration.**
